# Desensitization protocol enabling pediatric crossmatch-positive renal transplantation: successful HLA-antibody-incompatible renal transplantation of two highly sensitized children

**DOI:** 10.1007/s00467-016-3489-z

**Published:** 2016-09-01

**Authors:** Anna M. Adamusiak, Jelena Stojanovic, Olivia Shaw, Robert Vaughan, Neil J. Sebire, Martin Drage, Nicos Kessaris, Stephen D. Marks, Nizam Mamode

**Affiliations:** 1 Department of Transplantation at Guy’s and St Thomas’ NHS Foundation Trust, Guy’s Hospital, Great Maze Pond, 6th Floor, Borough Wing, SE1 9RT London, UK; 2 Department of Paediatric Nephrology at Great Ormond Street Hospital for Children NHS Foundation Trust, London, UK; 3 Clinical Transplantation Laboratory at Guy’s and St Thomas’ NHS Foundation Trust, London, UK

**Keywords:** Pediatric, Transplantation, Antibody-incompatible HLA, Desensitization

## Abstract

**Background:**

Renal transplantation improves quality of life (QoL) and survival in children requiring renal replacement therapy (RRT). Sensitization with development of a broad-spectrum of anti-HLA antibodies as a result of previous transplantation or after receiving blood products is an increasing problem. There are no published reports of desensitization protocols in children allowing renal transplantation from HLA-antibody-incompatible living donors.

**Methods:**

We adopted our well-established adult desensitization protocol for this purpose and undertook HLA antibody-incompatible living donor renal transplants in two children: a 14-year-old girl and a 13-year-old boy.

**Results:**

After 2 and 1.5 years of follow-up, respectively, both patients have stable renal allograft function despite a rise in donor-specific antibodies in one case.

**Conclusions:**

HLA-incompatible transplantation should be considered in selected cases for sensitized children.

## Introduction

Children with end-stage kidney disease (ESKD) on dialysis have worse outcomes compared with those transplanted [1, 2]. Dialysis has detrimental effects on children’s growth and cognitive development [3, 4]. Many national and international kidney allocation schemes prioritize children on deceased-donor waiting lists to ensure short waiting times [5, 6]. The median waiting time for a pediatric patient in the UK is 339 [95 % confidence interval (CI) 263–415] days [7].

The calculated HLA antibody reaction frequency (cRF) defines the level of sensitization and is derived from the proportion of the last 10,000 deceased donors exhibiting HLA antigens that react with a patient’s serum. In the UK, the median waiting time of a highly sensitized (cRF ≥ 85 %) child is 1241 (95 % CI 836–1646) days, which is no longer advantageous over the adult median waiting time of 1160 (95 % CI 1136–1184) days [7]. Highly sensitized children are also unlikely to receive kidneys via national living donor kidney sharing schemes as they are usually sensitized to common HLA antigens.

HLA-antibody-incompatible renal transplantation means that the organ expresses HLA antigens to which the recipient has pre-formed antibodies, resulting in a positive flow cytometric or cytotoxic crossmatch. Desensitization aims to remove sufficient antibodies to ensure a lack of reactivity of recipient serum to donor tissue (negative crossmatch). Living-donor HLA-antibody-incompatible renal transplantation provides a significant survival benefit for highly sensitized adult patients over those remaining on dialysis [8]. The only desensitization strategies in children reported in the literature have aimed at reducing their cRF while on the waiting list. A few cases have been described in which this approach has allowed transplantation of a child with a deceased donor kidney [9, 10].

There are no published reports of living-donor HLA-antibody-incompatible kidney transplants in children. It may be that the perceived risks and lack of experience in antibody removal in children has made pediatric centers reluctant to perform such transplants. We introduced our well-established adult desensitization protocol [11] to the pediatric transplant unit and undertook HLA-incompatible living-donor renal transplantation in two children. Our protocol is based on a test plasma exchange (PEX) to assess feasibility and estimate the number of sessions of antibody removal required to achieve a negative crossmatch prior to transplant. After the transplant, antibody removal is not performed for high levels of donor-specific antibodies (DSA) without accompanying deterioration in graft function or biopsy-proven rejection.

## First case

A 14-year-old white girl with ESKD secondary to congenital anomalies of the kidney and urinary tract was referred to our center from another European country. Two years previously, she had received a pre-emptive renal transplant from a deceased donor, which was complicated by renal vein thrombosis and graft nephrectomy within 24 h of implantation. After the first transplant, the patient developed anti-HLA antibodies with multiple specificities and a cRF of 99 %. The only potential living donor was her father, but the crossmatch at the referring center was positive. The patient was therefore entered into the National Living Donor Kidney Sharing Scheme (NLDKSS) but was unsuccessful after two runs.

An initial B- and T-cell-flow crossmatch against her father was positive, with 4.23 and 2.29 relative mean fluorescence (RMF), respectively. Reactivity was due to an antibody specific for mismatches HLA-B7 and -DQ8. Neither of these were repeat mismatches from her first transplant. To assess the feasibility of antibody removal, the patient underwent a test PEX. This reduced the level of her DSA, as measured with B7- and DQ-coated beads by Luminex assay, from 6854 to 2181 mean fluorescence intensity (MFI) and from 9346 to 4299 MFI, respectively. Both B- and T-cell-flow crossmatch were negative after test PEX, with a decrease in RMF to 1.63 and 1.2, respectively. Based on these results, we estimated that one session of antibody removal would be enough to achieve a negative crossmatch.

Tacrolimus and mycophenolate mofetil (MMF) were commenced 1 week prior to transplantation. Our patient underwent one session of plasma exchange on the day prior to her surgery, which reduced DSA from 11602 to 4625 MFI, following which a dose of immunoglobulin IV (IVIG) 0.5 g/kg was administrated overnight to prevent antibody rebound. On the day of surgery, the total DSA MFI was 8333, and B- and T-cell-flow crossmatches were negative (2 and 1.15 RMF, respectively). Induction therapy was with anti-thymocyte globulin (ATG) (1.5 mg/kg for 4 days), and maintenance immunosuppression comprised MMF, tacrolimus, and prednisolone. Prednisolone was tapered to 10 mg once daily at 6 weeks.

**Fig. 1:**
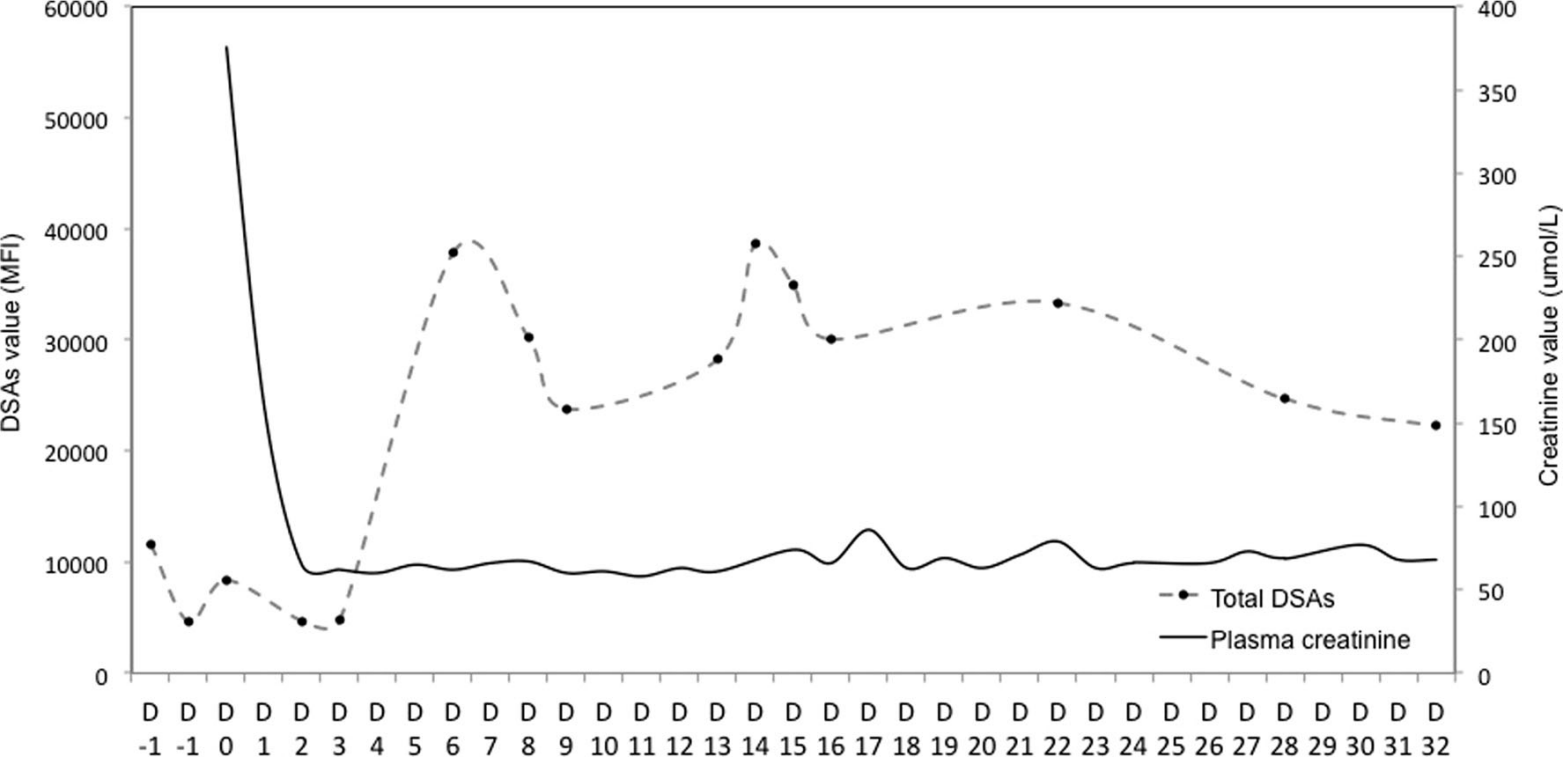
Posttransplant course of the first pediatric HLA-incompatible living-donor renal transplant in UK. Donor-specific antibodies and plasma creatinine during the first month. *PEX* plasma exchange, *TX* transplant, *BX* biopsy, *D* day posttransplant

The patient made a good recovery, with plasma creatinine improving until day 14, at which point it started to rise. This prompted administration of one dose of methylprednisolone IV prior to biopsy; biopsy showed no evidence of acute rejection despite rise in DSA. Plasma creatinine subsequently improved with hydration IV and one dose of methylprednisolone (Fig. 1). A further biopsy was performed on day 48 because of persistently raised DSA. There was no evidence of antibody-mediated rejection according to 2013 Banff criteria in either biopsy (t0, v0, i0, g0, ti0, ci0, ct0, mm0, cv1, ah1, ptc0) [12]. C4d staining was positive in the peritubular capillaries and glomeruli on both biopsies.

DSA levels fluctuated during the follow-up period, reaching values as high as 20,486 MFI for B7 and 19,222 MFI for DQ8 (Fig. 1). As per our protocol, in the absence of renal allograft dysfunction or histological evidence of rejection, we avoided antibody removal posttransplant. A protocol biopsy at 7 months posttransplant showed chronic changes of tubular atrophy and interstitial fibrosis (∼20 %), along with chronic vascular changes but no evidence of acute rejection and negative C4d staining (t0, v0, i0, ti0, g0, ci1, ct1, mm0, cv2, ah1, ptc0). Two years after transplant, the patient’s renal function remained stable, with an estimated glomerular filtration rate (eGFR) of 54 ml/min/1.73 m^2^ and a reduction in total donor-specific antibodies to 1499 MFI (B7 629; DQ8 870). The only infectious complication was a mild upper respiratory tract infection successfully treated orally with penicillin. The patient feels well and reports an improved quality of life (QoL) and has returned to school.

## Second case

A 13-year-old boy with ESKD due to solitary dysplastic kidney was referred to our center from the Middle East. He had been on hemodialysis for 10 months after renal allograft failure of his first living-donor renal transplant, which was performed 9 years earlier elsewhere and removed 6 months prior to retransplantation. He also had anti-HLA antibodies with multiple specificities and a cRF of 99 %. He was not eligible for deceased donation in the UK, but his mother did wish to donate to him.

The total MFI of DSA (A23, Cw7, DQB1*06:02, DP1) was 22,545; B-cell crossmatch was positive, with an RMF of 3.24. A test PEX showed a reduction in RMF to 2.45 and a fall in DSA to 15,321. Therefore, two sessions of double-filtration plasmapheresis (DFPP) were performed prior to his transplant, resulting in a B-cell-flow crossmatch of 1.68 RMF. IVIG at 0.5 g/kg was administered after the final session. His immunosuppression protocol was the same as described in the first case above, with the exception of alemtuzumab given at induction instead of ATG. ATG was used for the purpose of T-cell depletion in the first case because at that time we were not aware of any convincing safety data regarding using alemtuzumab in children. We had had experience in using ATG in pediatric recipients for treatment of severe rejection. Subsequently, data regarding the safety of alemtuzumab in children became available to us, and decided to use it instead of ATG for the second case.

This patient made an uneventful recovery from his surgery. A protocol biopsy at 2 months showed no features of rejection (t0, v0, i0, g0, ci0, ct0, cg0, mm0, cv1, ah1). C4d staining was diffusely positive in glomeruli but negative in peritubular capillaries. Plasma creatinine rose soon after biopsy following hospital admission for viral gastroenteritis. The lowest eGFR in that period was 24 ml/min/1.73 m^2^, which returned to baseline after empirical treatment with three doses of methylprednisolone IV. There was no associated rise in DSA, and two repeat biopsies showed no rejection with negative C4d staining. DSA—last measured 7 months posttransplant—was 18,745 MFI (A23 2602 MFI, Cw7 5893 MFI, DQB1*06:02 7112 MFI, DP1 3138 MFI). Latest (fourth) biopsy showed chronic changes (cv1 and ah1 only) and no features of acute rejection. Follow-up stood at 1 year at this report, with eGFR of 55 ml/min/1.73 m^2^.

## Discussion

We undertook successful patient desensitization enabling renal transplantation of two highly sensitized children from their related HLA-antibody-incompatible living donors.

There are proven benefits for a child having a kidney transplant rather than staying on dialysis, especially during puberty and adolescence [1–3, 13]. Both peritoneal dialysis and hemodialysis are associated with a worse QoL and an unsatisfactory growth rate [3, 14]. Allocation policies prioritizing children on the deceased donor waiting list have contributed to lower waiting times for a transplant. Sensitization status in children is, however, associated with a decreased rate of retransplantation after failure of the first graft [15]. Therefore, alternative solutions should be sought if sensitization status hinders transplantation of a child.

Renal transplantation from the HLA-antibody-incompatible living donors have not been undertaken so far in pediatric recipients, perhaps due to uncertainty about long-term outcomes and the lack of well-established desensitization protocols in children. Long-term outcomes of living-donor renal transplants in children are superior to transplants from deceased donors. The failed first kidney transplant from a deceased donor does not negatively influence the outcome of the second transplant from the living donor [16]. The long-term outcomes of HLA-incompatible renal transplants in adults are superior to remaining on dialysis [8], and expansion of pediatric HLA-antibody-incompatible transplantation will allow assessment of this in children.

Experience with ABO-incompatible pediatric renal transplantation provided the basis for optimizing antibody removal procedures in pediatric transplant recipients requiring antibody removal in the perioperative period or for treating antibody-mediated rejection [17–19]. There are recognized side effects of plasma exchange, such as clotting disturbances, hypoalbuminemia, and fluid shifts into the interstitial space, but these usually do not extend beyond a few days after antibody removal session and can be appropriately managed. Extracorporeal Immunoadsorbtion with columns specific for Fc antibody fragment has several advantages over conventional plasma exchange: It allows processing of higher volumes of plasma during one session and therefore efficient and specific anti-HLA antibody removal without loosing important plasma proteins, such as anticoagulation factors. We use immunoadsorption columns for our adult antibody-incompatible program but have reserved it for patients who require multiple sessions of antibody removal. This is because the effect on coagulation and fluid shifts are problematic in this situation. Normally, we prefer plasma exchange or double-filtration plasmapheresis, as this is a cheaper alternative compared with immunoadsorption. If no more than two plasma exchange sessions are required to achieve negative crossmatch as estimated by PEX, then benefits from immunoadsoption are questionable.

ATG was used for the first HLA-antibody-incompatible pediatric transplant and alemtuzumab for the second. We are planning to continue with the latter as an induction agent of choice. The use of ATG at induction limits options for treatment of severe rejection, which is not uncommon following HLA-antibody-incompatible transplants. We therefore advocate using alemtuzumab rather than ATG. There is no evidence that either of these agents is superior to another regarding long-term outcomes (rejection rate; incidence of infectious complications and malignancy; graft and patient survival), and therefore, the use of ATG is not arguable, especially as in some transplant centers alemtuzumab is not available for kidney transplant recipients.

There is debate about whether a more reactive immune system in children puts them at a higher risk of acute rejection. Acute and chronic antibody-mediated rejection leads to reduced renal allograft survival in HLA-antibody-incompatible renal transplantation [11, 20]. Desensitization and aggressive immunosuppression raises concerns about the risk of infective complications. In our cases, antibody-mediated rejection was absent even in the presence of DSA. We made similar observations in some of our adult patients with high levels of DSA, where possibly a process of accommodation takes place [21, 22].

Preventing sensitization in pediatric patients requiring dialysis remains paramount. There has been an increase in the percentage of sensitized patients on the waiting list due to both their previous transplants and frequent blood transfusions [7]. Transfusion of blood products represents a potentially avoidable source of sensitization. In a study by Scornik et al., transfusions induced sensitization in up to 35 % of patients aged 5–20 years compared with 7.5 % of patients >20 years [23, 24]. As development of anti-HLA antibodies due to blood transfusion is greater in children and decreases with advancing age, avoidance of pretransplant transfusion should be practiced. Prior transfusion is a proven risk factor for kidney graft loss in children [2].

The place of living-donor HLA-antibody-incompatible renal transplantation in children is ill defined. In our opinion, the kidney-sharing scheme should always be explored first because it gives the patient the chance of receiving a well-matched living-donor kidney with an immunologically low-risk transplant on a standard immunosuppressive regimen. However, in the broadly sensitized child, multiple runs in the sharing scheme are unlikely to yield results. Since the introduction of NLDKSS in the UK in January 2012, only one child has matched thus far, and this was with an ABO-incompatible donor [25].

**Fig. 2:**
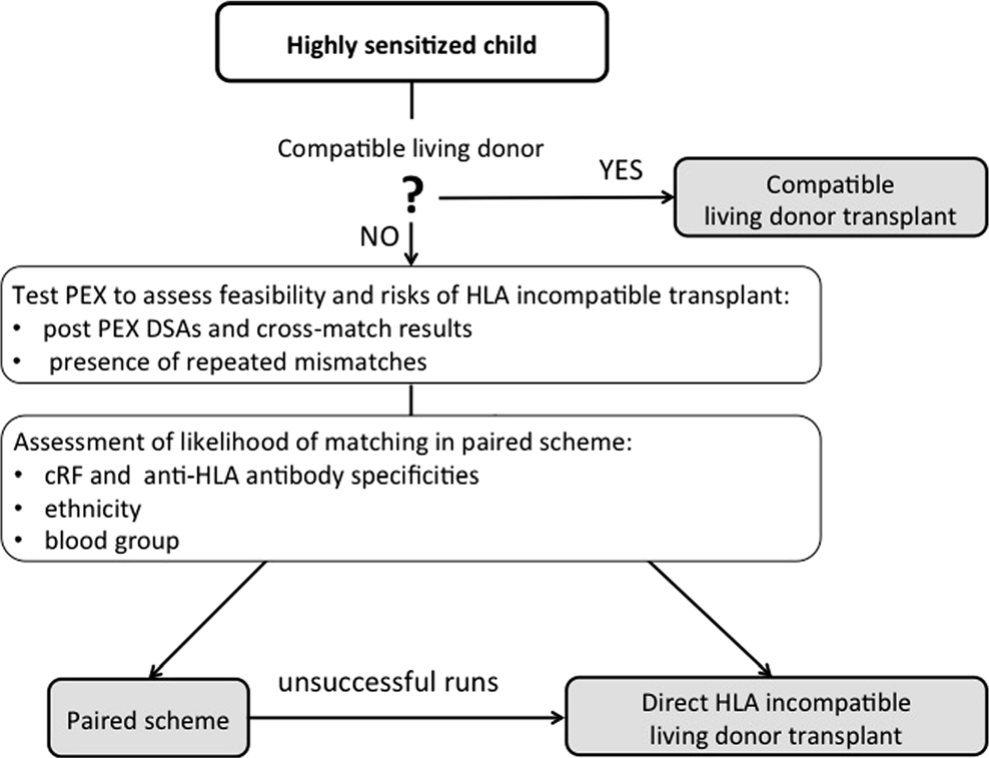
Algorithm supporting decision-making process as to whether to consider HLA-incompatible renal transplantation in a child. *PEX* plasma exchange, *DSAs* donor-specific antibodies, *cRF* calculated reaction frequency

In rare circumstances, a highly sensitized child has an HLA-compatible living donor. If this is not the case, the option of HLA-incompatible living-donor transplant should be explored. Our practice is to perform a test plasma exchange to assess feasibility for HLA-incompatible transplant. We measure how antibody removal can reduce the flow crossmatch and the level of donor-specific antibodies. Presence of repeated mismatches with the previous graft puts the patient at higher immunological risk of rejection. We offer the national living donor sharing scheme for all our highly sensitized children from the UK. Not all families are keen to participate, and for many patients, it is unlikely to have a match due to the broad sensitization, ethnicity, and type of blood group. These issues are discussed with the family to assist them in their decision-making process. After a few unsuccessful runs, we consider an HLA-incompatible direct transplant. We developed an algorithm supporting the decision-making process for sensitized children who are referred to our center (Fig. 2). Direct HLA-incompatible renal transplantation can be offered when an alternative HLA-compatible living donor is not available, cRF is >85 %, and results of a test PEX confirm its feasibility. Risk stratification is performed on an individual basis, as high DSA without broad sensitization (cRF 30–85 %), and certain donor and recipient blood group combinations would facilitate matching in the sharing scheme and make a direct HLA-antibody-incompatible transplant a less attractive alternative.

Although we advocate the attempt of matching a pediatric recipient with a compatible donor through paired exchange, this option is not available for children in many countries. In these circumstances, desensitization and HLA-antibody-incompatible renal transplantation would be the only chance for a highly sensitized child to receive a kidney from a living donor. Additionally, apart from experiencing long waiting times for deceased-donor kidney due to level of sensitization, children in some countries might not have this option at all. An example would be Japan, where deceased donation is still rare due to cultural traditions. Again, this highlights a demand in the transplant community for a desensitization protocol enabling HLA-antibody-incompatible renal transplantation of highly sensitized pediatric recipients.

In summary, our work provided evidence that pediatric HLA-antibody-incompatible renal transplantation is feasible and should be considered in certain circumstances.
